# Rank-One and Transformed Sparse Decomposition for Dynamic Cardiac MRI

**DOI:** 10.1155/2015/169317

**Published:** 2015-07-12

**Authors:** Xianchao Xiu, Lingchen Kong

**Affiliations:** Department of Applied Mathematics, Beijing Jiaotong University, Beijing 100044, China

## Abstract

It is challenging and inspiring for us to achieve high spatiotemporal resolutions in dynamic cardiac magnetic resonance imaging (MRI). In this paper, we introduce two novel models and algorithms to reconstruct dynamic cardiac MRI data from under-sampled *k* − *t* space data. In contrast to classical low-rank and sparse model, we use rank-one and transformed sparse model to exploit the correlations in the dataset. In addition, we propose projected alternative direction method (PADM) and alternative hard thresholding method (AHTM) to solve our proposed models. Numerical experiments of cardiac perfusion and cardiac cine MRI data demonstrate improvement in performance.

## 1. Introduction

Dynamic magnetic resonance imaging (MRI) reconstructs a temporal series of images to resolve the motion or the variation of the imaged object. It is often used in cardiac, perfusion, functional, and gastrointestinal imaging. The dynamic contrast variations in the myocardium are typically imaged 40–60 s in cardiac perfusion imaging; however, most patients cannot maintain a breath-hold for such long durations. The data in MRI, samples in *k* − *t* space of the spatial Fourier transform of the object, are acquired sequentially in time. Therefore, achieving high spatiotemporal resolutions is challenging and inspiring in dynamic MRI due to the hardware limitations and the risk of peripheral nerve stimulation.

Compressed sensing (CS) [1, 2] has been successfully applied to accelerate MRI, for example, [3–7] and references therein. Lustig et al. [3] systematically exploited the sparsity which is implicit in MRI and developed a framework for using CS in MRI. Liang [4] utilized low-rank matrix completion to dynamic MRI by considering each temporal frame as a column of a space-time matrix. Lingala et al. [5] and Zhao et al. [6] combined CS and low-rank matrix completion which finds a solution that is both low-rank and sparse. Gao et al. [8] proposed a different method which decomposes dynamic MRI matrix as a superposition of a low-rank component and a sparse component. It is worth noting that the low-rank component can model the temporally correlated background and the sparse component can model the dynamic information that lies on the top of the background. Otazo et al. [7] presented low-rank and sparse reconstructions for dynamic MRI using joint multicoil reconstruction for Cartesian and non-Cartesian *k*-space sampling.

Most of the above work is concerned about convex relaxation; then it comes to an interesting question whether we can deal with dynamic MRI using *ℓ*_0_-minimization. Many authors have made great effort; see [9–13], for example. Blumensath and Davies [9] were the first to propose the iterative hard thresholding (IHT) to solve a type of *ℓ*_0_-regularized problems and showed that the IHT method converges to a local minimizer. Beck and Eldar [10] introduced and analyzed three kinds of necessary optimality conditions for *ℓ*_0_-constrained problems. Bahmani et al. [14] studied *ℓ*_0_-constrained optimization in cases where nonlinear models are involved or the cost function is not quadratic. Recently, Lu and Zhang [11] presented a penalty decomposition method for solving a more general class of *ℓ*_0_-constrained and *ℓ*_0_-regularized minimization problems. Lu [12] considered *ℓ*_0_-regularized convex cone problems and then gave an IHT method and its convergence. More recently, Pan et al. [13] provided the first- and second-order necessary and sufficient optimality conditions for *ℓ*_0_-constrained optimization.

Motivated by the recent advances in the use of CS in MRI and *ℓ*_0_-minimization, we propose rank-one and transformed sparse models and algorithms to significantly accelerate dynamic MRI. In contrast to classical low-rank and transformed sparse decomposition, this paper has the following merits:

- We introduce the use of rank-one matrix in dynamic cardiac MRI data which avoids singular value decomposition (SVD).
- We assume the dynamic component has a sparse representation in some known sparsifying transformation, not itself.
- We develop new *ℓ*_0_-constrained model and *ℓ*_0_-regularized model, rather than their convex relaxation.
- We design two ADM-based algorithms, projected alternative direction method (PADM) and alternative hard thresholding method (AHTM). Numerical experiments of dynamic cardiac MRI illustrate the efficiency of our proposed algorithms.

The remainder of this paper is organised as follows.  Section 2 reviews some existing related work and proposes our new models.  Section 3 describes PADM and AHTM to solve our proposed models.  Section 4 reports experimental results.  Section 5 concludes this paper with some final remarks.

## 2. Model Analysis

We begin this section by introducing the low-rank and transformed sparse decomposition in [7]; that is,

(1) min L∗+βTS1s.t. EL+S=d,

where ‖·‖_*∗*_ is defined as the sum of all singular values and ‖·‖_1_ is defined as the sum of absolute values of all entries. *β* ∈ *ℝ* is a real positive weighting parameter to balance the weights of rank and sparsity, and *d* is the undersampled *k* − *t* data. *L* ∈ *ℂ* ^*m*×*n*^ is a low-rank matrix, which can model the temporally correlated background. *S* ∈ *ℂ* ^*m*×*n*^ is a sparse matrix, which can model the dynamic information that lies on top of the background. Generally, the dynamic component *S* has a sparse representation in some known sparsifying transformation *T* (e.g., temporal frequency domain), hence the idea of minimizing *TS* and not *S* itself. For acquisition with multiple receiver coils, *E* is given by the frame-by-frame multicoil encoding operator, which performs a multiplication by coil sensitivities followed by a Fourier transform according to the sampling pattern.

Compared with a low-rank or sparse constraint, (1) can significantly increase compressibility of dynamic MRI data and thus enable high acceleration factors. However, (1) is the convex relaxation problem. The original optimization problem associated with (1) is formulated as follows:

(2) min rank⁡L+βTS0s.t. EL+S=d,

and its low-rank and sparse constrained optimization problem is as follows:

(3) min EL+S−d2s.t. rank⁡L≤r TS0≤s,

where *r* denotes the rank of *L*, *s* denotes the sparsity of *TS*, and ‖·‖_0_ is *ℓ*_0_-norm, which counts the number of nonzero entries.

Note that if *E* and *T* reduce to identity matrix, (3) becomes a low-rank and sparse constrained optimization problem:

(4) min L+S−D2s.t. rank⁡L≤r S0≤s,

where *D* = *E* ^*∗*^ *d* is given data. Zhou and Tao [15] developed GoDec method to estimate the low-rank part *L* and the sparse part *S* in (4). GoDec alternatively updated *L* by SVD and *S* by hard thresholding. To overcome the difficulty of SVD, they proposed bilateral random projections. Even so, it still costs much time.

We find sometimes that the background is exactly a rank-one matrix, instead of a general low-rank matrix, so Li et al. [16] proposed to replace low-rank matrix with rank-one matrix which avoids any SVD completely. Xiu and Kong [17] extended it to the case of tensor decomposition and showed that it performs well in surveillance video. Specifically speaking, we set *L* = *u*1^*T*^, where *u* ∈ *ℂ* ^*m*^ and 1 denotes the vector in *ℝ* ^*n*^ whose entries are all 1, which leads to the following rank-one and transformed sparse matrix decomposition problem:

(5) min Eu1T+S−d2s.t. TS0≤s,

and its unconstrained version

(6) $$\begin{array}{c} \min{\left\|\;E\left(\;u1^{T}+S\;\right)-d\;\right\|}^{2}+\lambda {\left\|\;TS\;\right\|}_{0}, \end{array}$$

where *λ* > 0 is a regularized parameter.

## 3. Algorithm and Convergence

In this section, we present the projected alternative direction method (PADM) for (5) and alternative hard thresholding method (AHTM) for (6). Then we discuss their convergent properties.

### 3.1. Projected Alternative Direction Method for (5)

Let us look at the optimization problem (5). Inspired by ADM-based methods, it is easy to divide (5) into three subproblems:

(7) uk=argminu⁡u1T+Sk−1−Mk−12,Sk=argminS⁡uk1T+S−Mk−12:TS0≤s,Mk=uk1T+Sk−E∗Euk1T+Sk−d,

where *M* ^*k*^ is obtained by enforcing data consistency, where the aliasing artifacts corresponding to the residual in *k* − *t* space data *E* ^*∗*^(*E*(*u* ^*k*^1^*T*^ + *S* ^*k*^) − *d*) are subtracted from *u* ^*k*^1^*T*^ + *S* ^*k*^. Here, we denote these subproblems in (7) as *u*-subproblem, *S*-subproblem, and *M*-subproblem, respectively.

The first *u*-subproblem has the solution

(8) $$\begin{array}{c} u^{k}=mean\left(\;M^{k-1}-S^{k-1}\;\right), \end{array}$$

where, for any *Z* ∈ *ℂ* ^*m*×*n*^, mean(·) ∈ *ℂ* ^*m*^ is defined as

(9) $$\begin{array}{c} {\left(\;mean\left(\;Z\;\right)\;\right)}_{i}=\frac{1}{n}\overset{n}{\underset{j=1}{\sum }}Z_{ij},\;i=1,2,\ldots ,m. \end{array}$$

Before establishing the solution of the second *S*-subproblem, we first give a proposition about sparse projection.

**Proposition 1 .**

Let *Ω* = {*S* : ‖*TS*‖_0_ ≤ *s*}. Then *ℙ*_*Ω*_(*S*) = *T* ^*∗*^(*TS*)_max⁡(*s*)_, where *ℙ*_*Ω*_(*S*) denotes all but *s* largest absolute value components of *S*.

**Proof**

Letting *Ω* = {*S* : ‖*TS*‖_0_ ≤ *s*}, we have

(10) PΩSargminTS0≤s⁡X−S2=argminTS0≤s⁡TX−TS2=argminY0≤s⁡TX−Y2=PBTS,

where *ℬ* = {*Y* : ‖*Y*‖_0_ ≤ *s*} = *Y*_max⁡(*s*)_ and the third equation is satisfied by introducing *Y* = *TS*. Then, it holds that

(11) PΩST∗TPΩS=T∗PBTS=T∗TSmax⁡s.

The proof is completed.

From Proposition 1, we derive the solution of *S*-subproblem:

(12) $$\begin{array}{c} S^{k}=P_{\Omega }\left(\;M^{k-1}-u^{k}1^{T}\;\right). \end{array}$$

Hence the scheme is summarized as the following projected alternative direction method (PADM).

In Algorithm 1, *M*-subproblem is obtained by enforcing data consistency, so the convergence properties can be analyzed by considering the iterations of *u*-subproblem and *S*-subproblem. Now we will present the convergence result of PADM as follows.

**Theorem 2 .**

Let {(*u* ^*k*^, *S* ^*k*^)} be the sequence generated by the above PADM, and (*u* ^*∗*^, *S* ^*∗*^) is an accumulation point of {(*u* ^*k*^, *S* ^*k*^)}. Then (*u* ^*∗*^, *S* ^*∗*^) is a local minimizer of (5).

**Proof**

Let *F*(*u*, *S*) = ‖*E*(*u*1^*T*^ + *S*) − *d*‖^2^, for all *S* ∈ *Ω*. Indeed, one can observe that

(13) Fuk+1,Sk≤Fu,Sk,Fuk,Sk+1≤Fuk,S,∀S∈Ω.

It follows that

(14) $$\begin{array}{c} F\left(\;u^{k+1},S^{k+1}\;\right)\leq F\left(\;u^{k+1},S^{k}\;\right)\leq F\left(\;u^{k},S^{k}\;\right). \end{array}$$

Hence, the sequence {*F*(*u* ^*k*^, *S* ^*k*^)} is nonincreasing. Since (*u* ^*∗*^, *S* ^*∗*^) is an accumulation point of {(*u* ^*k*^, *S* ^*k*^)}, there exists a subsequence *K* such that lim_*k*∈*K*→*∞*_(*u* ^*k*^, *S* ^*k*^) = (*u* ^*∗*^, *S* ^*∗*^). We then observe that {*F*(*u* ^*k*^, *S* ^*k*^)}_*k*∈*K*_ is bounded, which together with the monotonicity of {*F*(*u* ^*k*^, *S* ^*k*^)} implies that {*F*(*u* ^*k*^, *S* ^*k*^)} is bounded below and hence lim_*k*→*∞*_(*u* ^*k*^, *S* ^*k*^) exists. This observation and the continuity of {*F*(*u* ^*k*^, *S* ^*k*^)} yield

(15) limk→∞⁡Fuk+1,Sk+1limk→∞⁡Fuk,Sk=limk∈K→∞⁡Fuk,Sk=Fu∗,S∗.

Using these relations and taking limits on both sides of (13) as *k* ∈ *K* → *∞*, we have

(16) Fu∗,S∗≤Fu,S∗,Fu∗,S∗≤Fu∗,S,∀S∈Ω.

In addition, from the definition of *Ω*, we know that ‖*TS* ^*k*^‖_0_ ≤ *s*, which immediately implies ‖*TS* ^*∗*^‖_0_ ≤ *s*. Thus, (*u* ^*∗*^, *S* ^*∗*^) is a saddle point of (5). Let Δ*S* be a variant such that *S* ^*∗*^ + Δ*S* ∈ *Ω* and let Δ*u* be a vector in *ℝ* ^*m*^. It then follows from (16) and the first-order optimality condition that [∇_*u*_ *F*(*u* ^*∗*^, *S* ^*∗*^)]^*T*^Δ*u* ≥ 0 and [∇_*S*_ *F*(*u* ^*∗*^, *S* ^*∗*^)]^*T*^Δ*S* ≥ 0. Henceforth, we obtain that

(17) Fu∗+Δu,S∗+ΔSFu∗,S∗+∇uFu∗,S∗TΔu+∇SFu∗,S∗TΔS≥Fu∗,S∗,

which implies that (*u* ^*∗*^, *S* ^*∗*^) is a local minimizer of (5).

### 3.2. Alternative Hard Thresholding Method for (6)

We can also divide the optimization problem (6) into the following *u*-subproblem, *S*-subproblem, and *M*-subproblem:

(18) uk=argminu⁡u1T+Sk−1−Mk−12,Sk=argminS⁡uk1T+S−Mk−12+λTS0,Mk=uk1T+Sk−E∗Euk1T+Sk−d.

Compared with the above subsection, we find that the *u*-subproblem and *M*-subproblem are the same; hereafter we mainly explain how to solve the *S*-subproblem. By applying the separability of the objective function and the technique of operator splitting, the *S*-subproblem can be converted into *mn* corresponding single variable minimization problems. Therefore, the following proposition will give the solution of the corresponding single variable minimization problem.

**Proposition 3 .**

Suppose that *x* ^*∗*^ is a solution of problem

(19) $$\begin{array}{c} \min\varphi_{t}^{\lambda }\left(\;x\;\right)=x^{2}-2tx+\lambda {\left|\;x\;\right|}_{0}, \end{array}$$

where *x* ∈ *ℝ* is variable and *t* ∈ *ℝ* is a parameter. Then,

(20) $$\begin{array}{c} x^{*}=H_{\lambda^{0.5}}\left(\;t\;\right)=\left\{\begin{array}{cc} t, & if\;\;\left|t\right|>\lambda^{0.5},\\ 0, & if\;\;\left|t\right|<\lambda^{0.5},\\ t\;\;or\;\;0, & otherwise. \end{array}\right. \end{array}$$

**Proof**

Note that *φ*_*t*_^*λ*^(0) = 0 and when *x* ≠ 0, *φ*_*t*_^*λ*^(*x*) = (*x* − *t*)^2^ + *λ* − *t* ^2^.

- If |*t*| > *λ* ^0.5^, *φ*_*t*_^*λ*^(*t*) < 0, then *x* ^*∗*^ = *t*.
- If |*t*| < *λ* ^0.5^, *φ*_*t*_^*λ*^(*t*) > 0, then *x* ^*∗*^ = 0.
- If |*t*| = *λ* ^0.5^, *φ*_*t*_^*λ*^(*t*) = 0, then *x* ^*∗*^ = *t* or 0.

Thus, we immediately obtain the proposition.

It is straightforward to establish the following result.

**Proposition 4 .**

For the general *ℓ*_0_-regularized problem,

(21) $$\begin{array}{c} \min{\left\|\;AX-B\;\right\|}^{2}+\lambda {\left\|\;X\;\right\|}_{0}, \end{array}$$

where *A* ∈ *ℝ* ^*p*×*m*^ is some transform matrix and *B* is a given matrix in *ℝ* ^*p*×*n*^. Then, the solution *X* ^*k*^ is described as follows:

(22) $$\begin{array}{c} X^{k}=H_{\lambda^{0.5}}\left(\;X^{k-1}-A^{T}\left(\;AX^{k-1}-B\;\right)\;\right), \end{array}$$

where *ℋ*_*λ*^0.5^_ is the hard thresholding operator defined as

(23) $$\begin{array}{c} H_{\lambda^{0.5}}\left(\;X_{ij}\;\right)=\left\{\begin{array}{cc} X_{ij}, & if\;\;\left|X_{ij}\right|>\lambda^{0.5},\\ 0, & if\;\;\left|X_{ij}\right|<\lambda^{0.5},\\ X_{ij}\;\;or\;\;0, & otherwise. \end{array}\right. \end{array}$$

**Proof**

The conclusion holds directly from Proposition 3 by separating the objective function.

From Proposition 4, the solution of *S*-subproblem is characterized as

(24) $$\begin{array}{c} S^{k}=T^{*}\left(\;H_{\lambda^{0.5}}\left(\;T\left(\;M^{k-1}-u^{k}1^{T}\;\right)\;\right)\;\right). \end{array}$$

Based on the above argument, we have derived the following alternative hard thresholding method (AHTM) for (6).

In the end of this subsection, we will establish the convergence result of Algorithm 2.

**Theorem 5 .**

Let {(*u* ^*k*^, *S* ^*k*^)} be the sequence generated by the above AHTM, and (*u* ^*∗*^, *S* ^*∗*^) is an accumulation point of {(*u* ^*k*^, *S* ^*k*^)}. Then (*u* ^*∗*^, *S* ^*∗*^) is a local minimizer of (6).

**Proof**

Let *f*(*u*, *S*) = ‖*E*(*u*1^*T*^ + *S*) − *d*‖^2^; then

(25) Fu,Sfu,S+λTS0=Eu1T+S−d2+λTS0.

Like the proof of Theorem 2, we derive that (*u* ^*∗*^, *S* ^*∗*^) is a saddle point of (6). We choose Δ*u* to be a vector in *ℝ* ^*m*^, and Δ*S* satisfies ‖*T*(*S* ^*∗*^ + Δ*S*)‖_0_ = ‖*TS* ^*∗*^‖_0_. Based on the convexity of *f*(*u*, *S*), we have [∇_*u*_ *f*(*u* ^*∗*^, *S* ^*∗*^)]^*T*^Δ*u* ≥ 0 and [∇_*S*_ *f*(*u* ^*∗*^, *S* ^*∗*^)]^*T*^Δ*S* ≥ 0. Using this relation, we obtain that

(26) Fu∗+Δu,S∗+ΔSfu∗,S∗+∇ufu∗,S∗TΔu+∇Sfu∗,S∗TΔS+λTS∗+ΔS0≥fu∗,S∗+λTS∗0=Fu∗,S∗.

Hence (*u* ^*∗*^, *S* ^*∗*^) is a local minimizer of (6).

## 4. Numerical Experiments

In this section, we conduct numerical experiments to compare the performance of IST [7], PADM, and AHTM for dynamic cardiac MRI data. We apply all these methods on MR images: cardiac perfusion and cardiac cine, which are available at the website http://cai2r.net/resources/software/ls-reconstruction-matlab-code. All experiments are implemented in MATLAB (MathWorks, Natick, MA) on a desktop computer with Intel Core I5 2.60 GHz CPU and 8 GB of RAM.

We now address the initialization and the termination criteria for these methods. In particular, we set the stopping criterion as RelErr < 2.5*E* − 3, where

(27) $$\begin{array}{c} RelErr≔\frac{{\left\|u^{k}1^{T}+S^{k}-\left(u^{k-1}1^{T}+S^{k-1}\right)\right\|}^{2}}{{\left\|u^{k-1}1^{T}+S^{k-1}\right\|}^{2}}. \end{array}$$

We choose *λ* = 0.01 in AHTM as suggested in [7]. However, we do not know the sparse parameter *s* beforehand. Hence, in this paper, we initialize *s* as one percent of pixels in each frame of *TS*.

### 4.1. Cardiac Perfusion

**Figure 1:**
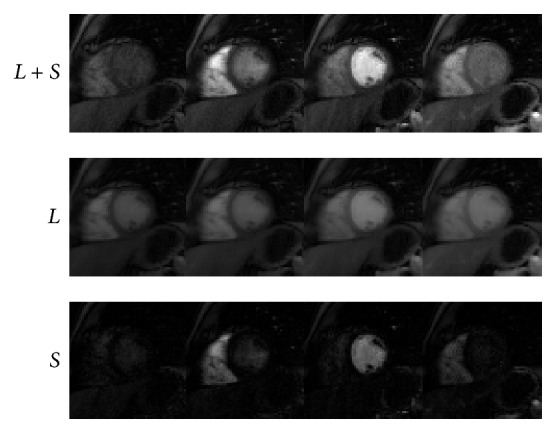
Performance of IST for dynamic cardiac perfusion.

**Figure 2:**
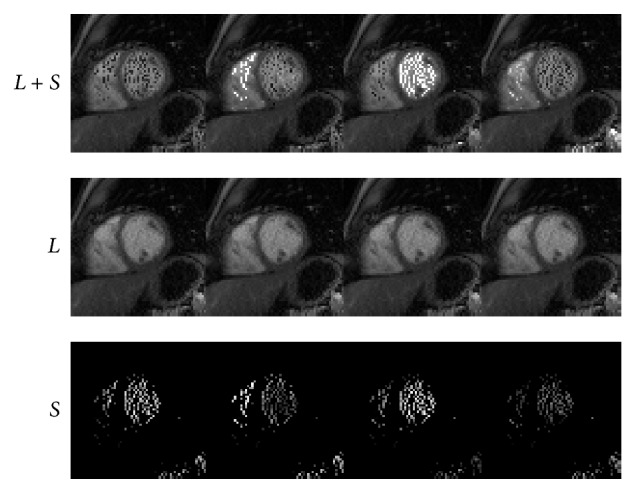
Performance of PADM for dynamic cardiac perfusion.

**Figure 3:**
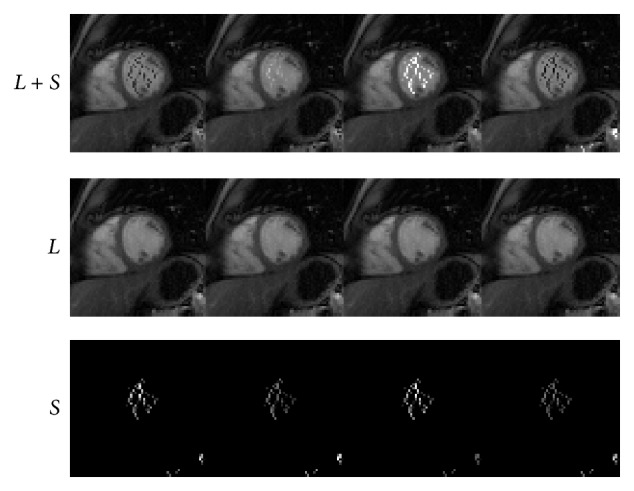
Performance of AHTM for dynamic cardiac perfusion.

We next conduct numerical experiments to test the performance of IST [7], PADM, and AHTM for dynamic cardiac perfusion MRI data. The computational results of the three methods are presented in Figures 1–3. In detail, we use *L* + *S* (the first row) to denote the original dynamic cardiac perfusion MRI frames, *L* (the second row) to denote the reconstructed background parts, and *S* (the third row) to denote the reconstructed moving parts. Comparing Figures 1–3, we can observe that PADM (see Figure 2) and AHTM (see Figure 3) are easier to recognize the pathological parts than IST (see Figure 1), which is due to the use of *ℓ*_0_ rather than its convex relaxation *ℓ*_1_. This capacity may be useful to identify lesions that are difficult to visualize in the original images.

In addition, Table 1 reports the computational results for dynamic cardiac perfusion MRI data. Here, the number of iterations is given in the second column (i.e., Iteration). The third column (i.e., Time) lists the computing time in seconds. The relative error is reported in the last column (i.e., RelErr). It is evident to see that the “Time” and “Iteration” of PADM and AHTM are lower than these of IST. This is because we employ rank-one to avoid SVD.

### 4.2. Cardiac Cine

**Figure 4:**
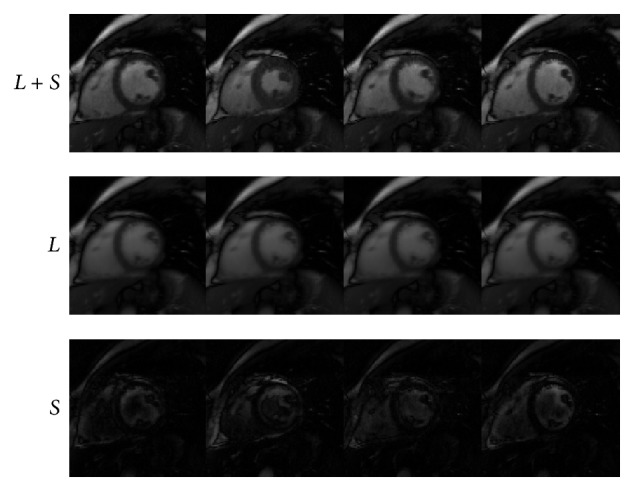
Performance of IST for dynamic cardiac cine.

**Figure 5:**
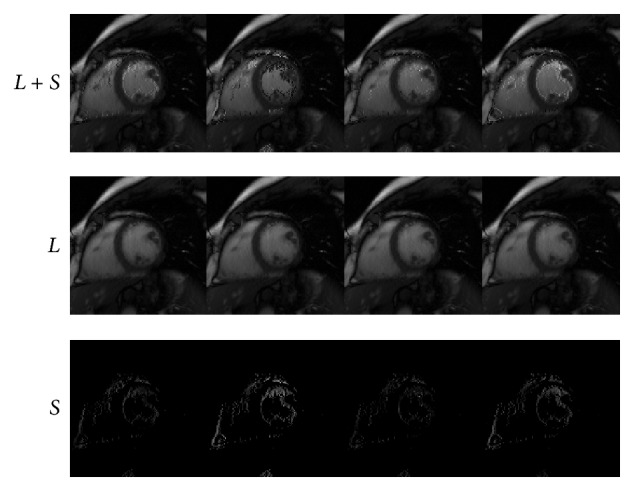
Performance of PADM for dynamic cardiac cine.

**Figure 6:**
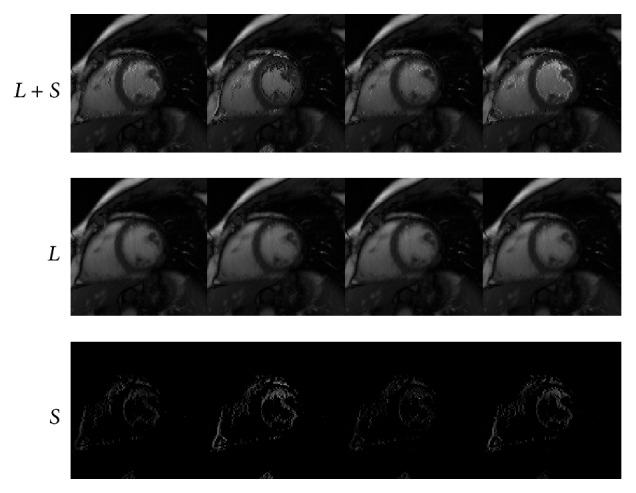
Performance of AHTM for dynamic cardiac cine.

Similarly, Figures 4–6 and Table 2 demonstrate the performance of IST [7], PADM, and AHTM for dynamic cardiac cine MRI data. From the third row, we find that Figures 5 and 6 can get clearer moving parts, because we can tune the sparsity of *s* and *λ*.

## 5. Conclusions

In this paper, we focused on the application of rank-one and transformed sparse decomposition for dynamic cardiac MRI. We established the projected alternative direction method and alternative hard thresholding method and showed their convergent results. Finally, numerical experiments demonstrated the efficiency and effectiveness of our proposed methods.

## Figures and Tables

**Algorithm 1:**
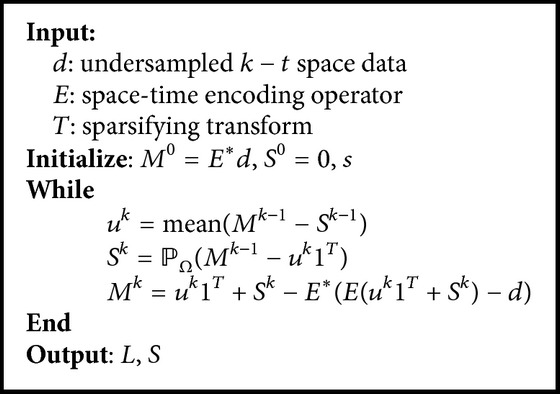
Projected alternative direction method for (5).

**Algorithm 2:**
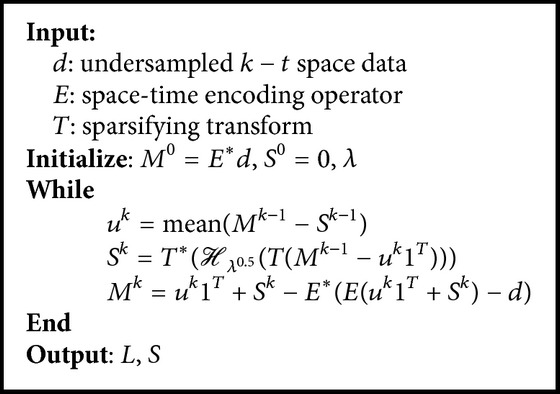
Alternative hard thresholding method for (6).

**Table 1:** Computational results for dynamic cardiac perfusion.

Algorithm	Iteration	Time	RelErr
IST	36	80.38	2.5*E* − 03
PADM	25	59.47	2.5*E* − 03
AHTM	31	63.25	2.4*E* − 03

**Table 2:** Computational results for dynamic cardiac cine.

Algorithm	Iteration	Time	RelErr
IST	26	140.13	2.4*E* − 03
PADM	18	81.49	2.5*E* − 03
AHTM	22	94.26	2.4*E* − 03
